# Is there any link between tumor-induced osteomalacia and psoriasis? A case report

**DOI:** 10.1186/s40200-017-0315-5

**Published:** 2017-08-23

**Authors:** Mojtaba Akbari, Bagher Larijani, Sasan Sharghi, Ali Jalili, Sayed Mahmoud Sajjadi-Jazi

**Affiliations:** 1 Students’ Scientific Research Center, Tehran University of Medical Sciences, Tehran, Iran; 2 Endocrinology and Metabolism Research Center, Endocrinology and Metabolism Clinical Sciences Institute, Tehran University of Medical Sciences, Tehran, Iran; 3 Diabetes Research Center, Endocrinology and Metabolism Clinical Sciences Institute, Tehran University of Medical Sciences, Tehran, Iran

**Keywords:** Tumor-induced osteomalacia, Psoriasis, Fibroblast growth factor-23

## Abstract

**Background:**

Tumor-induced osteomalacia is an uncommon paraneoplastic syndrome caused by Fibroblast growth factor-23-secreting tumors. It is characterized by phosphaturia, hypophosphatemia, and a high plasma level of alkaline phosphatase.

**Case presentation:**

We report a young patient with psoriasis who had suffered from bone pain and muscle weakness for more than 6.5 years. He was finally diagnosed with tumor-induced osteomalacia. However, mistakenly attributing the patient’s signs and symptoms to psoriatic arthritis for a long time had resulted in multiple complications for the patient. Finally, the tumor was localized and surgically resected. This resulted in clinical improvements and the resolution of all biochemical abnormalities.

**Conclusion:**

To our knowledge, this is the second case of tumor-induced osteomalacia accompanied by psoriasis. There is growing evidence to suggest that Fibroblast growth factor-23 has a role in regulating immune function while an increased level of it may play a role in the pathogenesis of psoriasis. As a result, tumor-induced osteomalacia may affect the psoriasis clinical course by secreting a high amount of Fibroblast growth factor-23. On the other hand, several studies have showed an increased risk of malignancy among patients with psoriasis. Consequently, long-term psoriasis may predispose patients to Fibroblast growth factor-23-secreting tumors. Finally, as psoriasis is a common disease, this presentation may simply be a coincidence.

## Background

Tumor-induced osteomalacia (TIO) is an uncommon paraneoplastic syndrome caused by Fibroblast Growth Factor-23 (FGF-23)-secreting tumors. It is characterized by hypophosphatemia, phosphaturia, increased level of alkaline phosphatase (ALP), low or normal 1, 25-dihydroxyvitamin D, normal serum calcium, and normal parathyroid hormone (PTH) level [1]. The disease usually presents itself with multiple bone fractures, chronic bone pain and muscle weakness. The diagnosis frequently is missed for many years, leading to several complications in patients [2, 3].

Psoriasis is a chronic autoimmune disease affecting about 2%–3% of the general population [4]. The main manifestation of psoriasis is skin involvement and Plaque-type psoriasis is the most common form. However, inflammatory processes can occur elsewhere and now it is considered as a multisystem chronic inflammatory disorder [4, 5]. The precise psoriasis etiology is not defined; however, several factors including genetic, environmental, hormonal, and immunologic issues contribute to its pathogenesis [4].

We report a young patient with psoriasis who had suffered from bone pain and muscle weakness for more than 6.5 years, and had become bedridden due to multiple fragility fractures. He was unsuccessfully treated for psoriatic arthritis for several years and was finally diagnosed with TIO.

## Case presentation

A 26 year-old man was referred to our center (Endocrinology ward, Shariati hospital, Tehran, Iran) because of multiple fragility fractures that had occurred to him within eight months before admission.

He had been treated with topical treatments for plaque-type psoriasis since he was 10. He was hospitalized 6.5 years ago in a rheumatologic ward due to muscle weakness, bone pain, low back pain, bilateral hip pain, and generalized psoriatic skin lesions. His workup at that time, including plain radiographs and magnetic resonance imaging (MRI), showed generalized skeletal demineralization without any fracture, as well as bilateral hip and sacroiliac joints signal change in favor of possible psoriatic arthritis. In addition, his laboratory tests revealed hypophosphatemia, high ALP and phosphaturia (Table 1). Due to generalized psoriasis skin lesions, which was uncontrolled by topical treatment, and probable psoriasis arthritis, a systemic therapy was initiated. The patient was discharged with the diagnosis of probable hypophosphatemic osteomalacia combined with psoriatic arthritis without a further evaluation for the cause of hypophosphatemia. He was prescribed to take methotrexate 15 mg weekly, sulfasalazine 1 g twice a day, folic acid 5 mg daily, prednisolone 5 mg daily, Indomethacin 100 mg daily, phosphate 500 mg twice a day, calcium carbonate 500 mg twice a day, and vitamin D 50000 international units (IU) weekly.

**Table 1:** Patient’s laboratory data

											24-h urine collection			
Laboratory data	Mg mg/dl	Ca mg/dl	P mg/dl	PTH pg/ml	25(OH) vit D	ALP IU/L	Cr mg/dl	FGF-23 kRU/L (26–110)	ESR mm/h	CRP mg/L	Ca mg/24 h	P mg/24 h	Cr mg/24 h	Ca mg/24 h
First admission (January 2010)	2.2	9.5	1.5	16	12.3 ng/ml	553	0.6		7	1	95	616	1050	95
second admission (July 2015)	1.7	9.5	1	22	106 nmol/L	744	0.7	458	5	4	193	378	990	193
Post operation (without phosphate supplementation)		9.1	4.9			345	0.85							

*kRU/L* kilo-Relative Units per liter, *ESR* erythrocyte sedimentation rate, *CRP* C-reactive protein

His symptoms, however, progressively worsened despite the treatment. After a year, he could barely walk without a cane, and later he had to use a walker and eventually became bedridden eight months before admission to our center. During these years, he had several visits to rheumatology clinics, but only his psoriatic arthritis medications and phosphate dose were changed. He was referred to our center due to osteomalacia, multiple bone fractures, and hypophosphatemia.

**Fig. 1:**
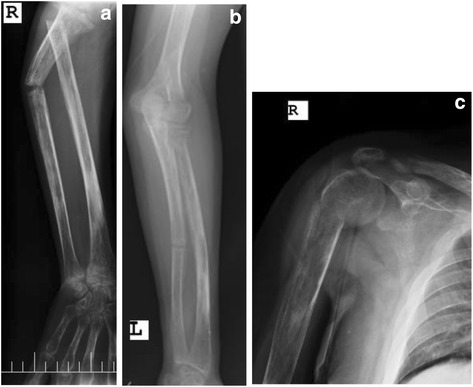
X-Ray radiographs showing severe osteomalacia and multiple fractures. **a** Right radial head and right ulnar shaft fracture; **b** Left mid ulnar shaft fracture; **c** Right humerus anatomical neck fracture and Distal clavicular resorption

**Fig. 2:**
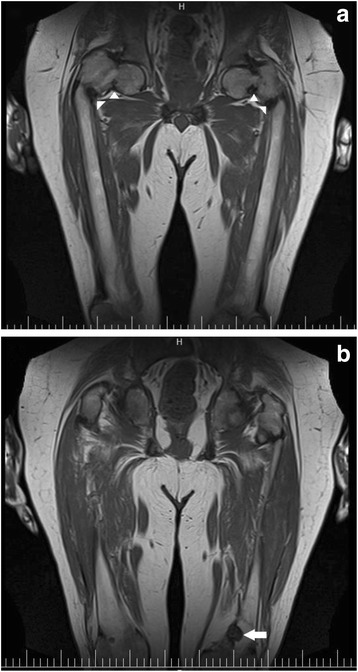
T1 weighted MRI: **a** Femoral neck and subtrochanteric fractures on both sides (arrow heads), and bilateral coxa vara deformities; **b** A mass-like lesion in medial aspect of the left distal femur metaphysis (arrow)

At the time of admission, the patient was bedridden and suffering from generalized bone pain. He estimated a 20-cm loss in his height. He had no other medical problems, was not a smoker, not a drinker, and had no relevant family history. He was taking methotrexate 17.5 mg weekly, folic acid 5 mg daily, phosphate 500 mg twice a day, calcium carbonate 500 mg twice a day and calcitriol 0.5 mg daily. The physical examination showed a decrease in both upper and lower limbs muscle force, multiple tender points due to fractures, kyphoscoliosis, and skin hyperpigmentation due to previous psoriatic skin lesions but with no active lesion. Joints examination did not show any sign of inflammation. Radiography studies showed osteomalacia, looser zones, and multiple fractures in ribs, neck of the humerus of both sides, both ulnas, the right radius, the proximal femur of both sides, and the left first metacarpal bone (Figs. 1 and 2a). A severe scoliosis and a biconcave appearance of multiple vertebral bodies without any obvious fracture were also noted. The sacroiliac and hip joints MRI did not show any evidence of inflammatory arthritis. Laboratory data showed isolated hypophosphatemia (Table 1) along with very high urine fractional excretion of phosphate (26.73%). The renal phosphate threshold normalized for the glomerular filtration rate was 0.73 mg per 100 ml (ml) (age- and gender- specific normal range: 3.09–4.18 mg per 100 ml [2]). Due to these evidences, we suspected an FGF-23-related hypophosphatemia and checked it, which was too high (Table 1). In the absence of any related family history and the presentation of disease in adulthood, acquired causes of FGF-23-mediated osteomalacia, particularly TIO, were suspected.

**Fig. 3:**
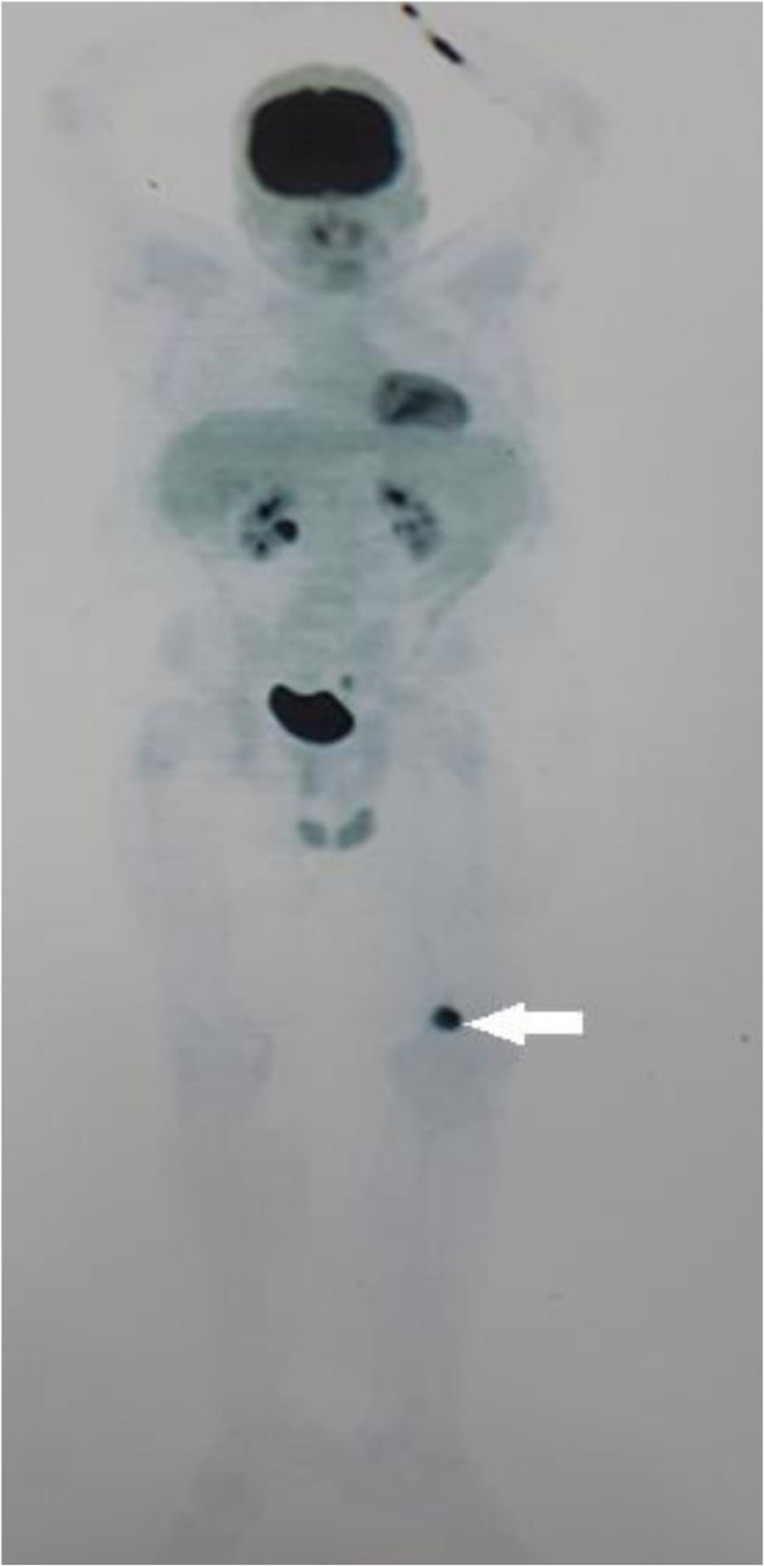
18F–FDG PET: a hypermetabolic lesion in medial aspect of the left distal femur (arrow)

**Fig. 4:**
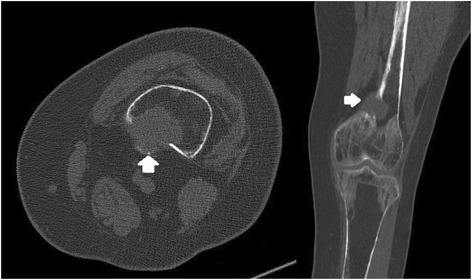
Knee CT scan: a 28*26*24 mm lobulated lytic mass in posteromedial aspect of the left distal femoral metaphysis with cortical disruption and extra osseous soft tissue formation (arrow)

To localize the tumor, a whole body MRI was performed, which revealed a mass-like lesion in the proximal metaphysis of the left tibia and the distal metaphysis of the left femur (Fig. 2b). The 18F–fluorodeoxyglucose positron emission tomography (FDG-PET) scan showed a hypermetabolic lesion (maximum standardized uptake value = 21.28) in the medial aspect of the left distal femur (Fig. 3). The Knee CT scan revealed a 28*26*24 mm lobulated lytic mass in the posteromedial aspect of the left distal femoral metaphysis with a cortical disruption and an extra osseous soft tissue formation (Fig. 4). Then an incisional biopsy of both lesions was performed.

**Fig. 5:**
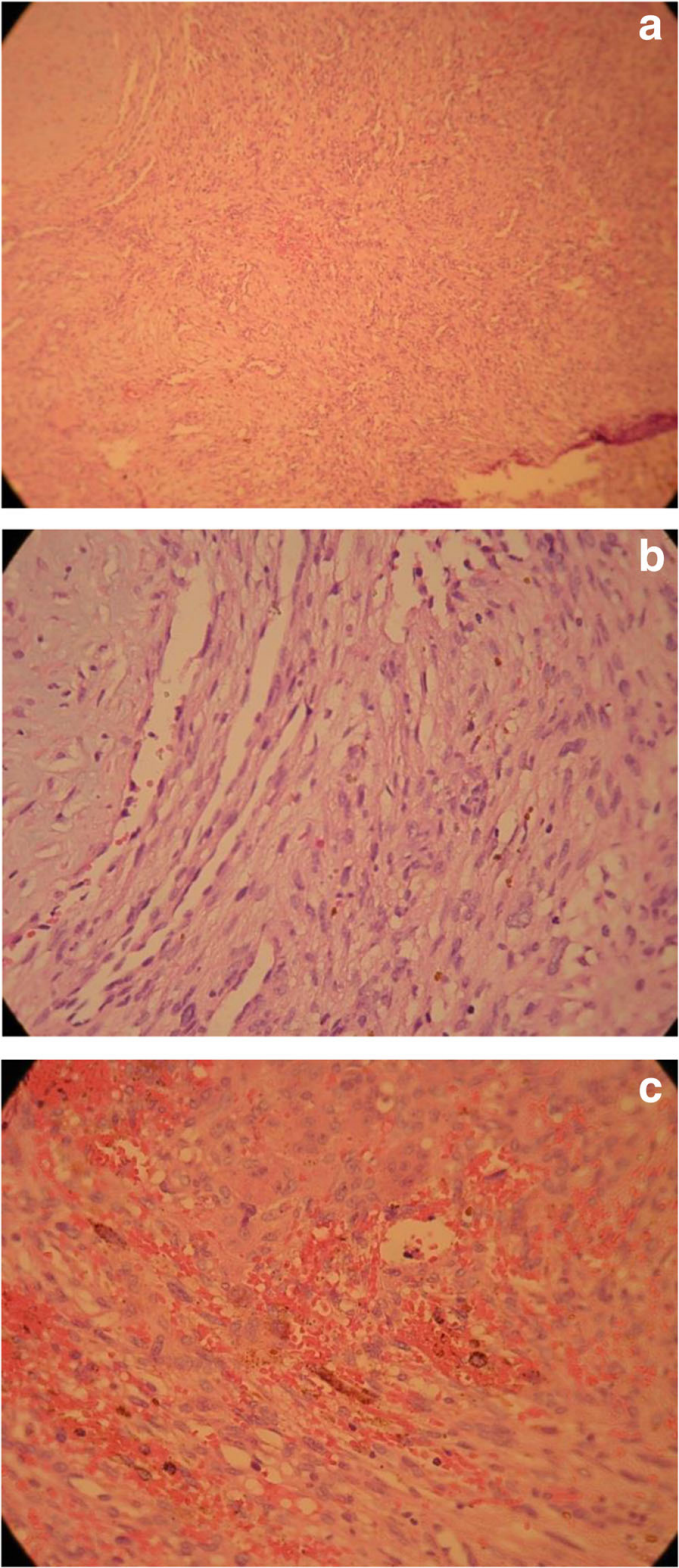
Hematoxylin and eosin stain of distal femur lesion compatible with phosphaturic mesenchymal tumor. **a** Benign-appearing hypervascular mesenchymal tumor (low power field); **b** bland-looking spindle cells with small nuclei and many small and few large vessels without staghorn feature (high power field); **c** Osteoclast like giant cells, Hemorrhagic stroma and hemosiderin-laden macrophages (high power field)

The histological examination of the distal femur lesion demonstrated a hypocellular neoplastic tissue composed of bland–looking spindle cells with small nuclei and indistinctive nucleoli. The tumor consisted of many small and a few large vessels without a staghorn feature. Some osteoclasts, like giant cells and many fibrohistiocytic cellular nests with hemorrhagic stroma and hemosiderin-laden macrophages were seen. Areas of matrix calcification and fat tissue were also noticed within the tumor. No atypia or mitotic figure was identified (Fig. 5). The immunohistochemistry (IHC) study was positive for vimentin in all cells and the cluster of differentiation (CD) 68 in some of them. IHC studies for S100, Epithelial membrane antigen (EMA), CD34, CD31, pan Cytokeratin (PCK) and Factor VIII-Related Antigen was negative and thus compatible with a phosphaturic mesenchymal tumor. The histological examination of the proximal tibia lesion showed only fragmented bone trabeculae with no malignancy.

Due to the presence of a severe hypophosphatemia, osteomalacia, and with respect to a high likelihood of fractures during surgery, the surgical resection of the tumor was postponed for two months, during which he was treated with phosphate 4 g daily in divided doses plus calcitriol 3 mg daily. Then the tumor was completely resected and a total knee arthroplasty was performed. The permanent pathology confirmed the findings of the incisional biopsy results. The phosphate supplementation was gradually tapered and discontinued, however, the serum phosphate increased to a normal range (Table 1). After considering the rheumatology and dermatology consultation, the dose of methotrexate was reduced to 7.5 mg per week. However, his psoriasis skin lesions remained in remission after the dose reduction.

In his follow up, muscle weakness and bone pain gradually improved and his fractures were managed by orthopedic service. After 6 months, he could ambulate with bilateral axillary crutches. Now the patient is scheduled for bilateral total hip replacement surgery.

## Discussion

FGF-23-secreting tumors usually originate from mesenchymal tissues [6]. About 70–80% of these tumors are phosphaturic mesenchymal tumors; mixed connective tissue variants and less common types are hemangiopericytoma, osteosarcoma, giant cell tumors etc. [7]. The tumors are usually small with a slow growth rate.

TIO is usually missed for many years due to the occult nature of the disease, its non-specific presentations, and the serum phosphate level being unchecked in early routine tests [2, 3]. In addition, non-specific signs and symptoms mislead physicians into more common diseases, especially rheumatologic ones such as rheumatoid arthritis and seronegative spondyloarthritis [3, 8]. In our case, mistakenly attributing the patient’s signs and symptoms to the psoriatic arthritis for a long time had resulted in multiple complications for the patient.

In this regard, clinical suspicion and routine laboratory investigation including measurement of plasma and urine phosphate levels in all patients with unexplained long-term muscle weakness or bone pain/fractures is prudent. If the urinary excretion of phosphate is high in spite of a low serum phosphate level, TIO should be included in the differential diagnosis, and if confirmed, a thorough investigation is needed to localize the tumor.

Even after diagnosis, locating the tumor is difficult [2, 9]. Nowadays, a multi-modality approach is recommended for tumor localization in patients. A whole body MRI, CT scan, ^111^Indium octreotide scintigraphy and PET scan using 18F–fluorodeoxyglucose or ^68^Ga-DOTATATE are possible options [2, 10–13].

The treatment of choice in these patients is the surgical resection of the tumor, which leads to a rapid normalization of biochemical abnormalities, remineralization of the bones and relief of the symptoms [2].

According to our literature review, this is the second case of TIO accompanying psoriasis. The first case was a 55-year-old Chinese man with TIO caused by an infratemporal fossa tumor. He had had a history of well-controlled psoriasis for 30 years before being diagnosed with TIO [14]. However, the clinical course and drugs that he used to control his psoriasis was not mentioned.

A recent published article by Okan et al. demonstrated that the FGF-23 level is high in psoriasis and an elevated FGF-23 is associated with psoriasis severity [15]. Besides the important role of FGF-23 in phosphate homeostasis, there is growing evidence to suggest that FGF-23 has a role in regulating immune function. An elevated FGF-23 concentration is associated with higher levels of inflammatory markers including inteleukin-6 (IL-6), C-reactive protein (CRP), fibrinogen, and tumor necrosis factor alpha (TNFα) [15, 16]. Several possible mechanisms can explain these findings. One possible explanation is that FGF-23 directly stimulates inflammation [16]. Another possibility is that FGF-23 can indirectly induce inflammation by decreasing the level of 1, 25-dihydroxyvitamin D. FGF-23 impairs the production of 1, 25-dihydroxyvitamin D and accelerates its degradation by inhibiting renal 1alpha-hydroxylase and stimulating 24-hydroxylase respectively [15, 16]. 1, 25-dihydroxyvirtamin D is an inhibitor of T-cell proliferation and other inflammatory mediators and also has a role in inhibiting keratinocyte proliferation and stimulating its differentiation [15–18]. Taken together, these findings suggest that an increased FGF-23 level may play a role in the pathogenesis of psoriasis.

Our patient psoriasis was controlled by topical treatment until his first admission to hospital at the rheumatology ward. In that time, due to severe and generalized psoriasis skin lesions and possible psoriatic arthritis, his medication was changed to methotrexate, sulfasalazine, prednisolone and Indomethacin. This psoriasis skin flare-up is in concordance with radiologic and laboratory evidence of osteomalacia and hypophosphatemia respectively and indicates the presence of TIO at that time. In his second admission to hospital when he was admitted in our center, his psoriasis was well-controlled but he received methotrexate, a potent anti-inflammatory drug. After tumor resection and with regard to the dermatology consultation, the dose of methotrexate was reduced but not discontinued. As a result, we could not evaluate the clinical course of psoriasis skin lesions after the methotrexate withdrawal. However, his psoriasis remained in remission after the dose reduction. Therefore, we hypothesized that TIO may alter the clinical course of psoriasis by secreting a high amount of FGF-23.

On the other hand, several studies have showed an increased risk for malignancy among patients with psoriasis [19–24]. Consequently, long-term psoriasis may predispose patients to FGF-23-secreting tumors. Finally, as psoriasis is a common disease, this presentation may simply be a coincidence.

## Conclusion

To our knowledge, this is the second case of TIO accompanied by psoriasis. There is growing evidence to suggest that FGF-23 has a role in regulating the immune function and an increased level of it may play a role in the pathogenesis of psoriasis. Therefore, TIO may affect the psoriasis clinical course by secreting a high amount of FGF-23. On the other hand, an increased risk of malignancy among patients with psoriasis is proven by neumerous investigations and consequently, long-term psoriasis may predispose patients to FGF-23-secreting tumors. Whether there is any association between these two diseases or this presentation is only a coincidence needs further investigations.

## Abbreviations

25(OH) vit D: 25-hydroxy vitamin D
^68^Ga-DOTATATE: ^68^Gallium-[1, 4, 7, 10-tetraazacyclododecane-N, N′, N′′, N′′′-tetraacetic acid]-D-Phe^1^, Tyr^3^-octreotate
ALP: Alkaline phosphatase
Ca: Calcium
CD: Cluster of differentiation
Cr: Creatinine
CRP: C-reactive protein
CT: Computed tomography
dl: deciliter
EMA: Epithelial membrane antigen
ESR: Erythrocyte sedimentation rate
FDG-PET: 18F–fluorodeoxyglucose positron emission tomography
FGF-23: Fibroblast growth factor-23
hrs: hours
IHC: Immunohistochemistry
IL6: Inteleukin-6
IU: International unit
kRU/L: kilo-Relative Units per liter
L: liter
Mg: Magnesium
mg: milligram
ml: milliliter
mm: millimeter
MRI: Magnetic resonance imaging
ng: nanogram
nmol: nanomol
P: Phosphorus
PCK: Pan Cytokeratin
pg: pictogram
PTH: Parathyroid hormone
TIO: Tumor-induced osteomalacia
TNFα: Tumor necrosis factor alpha
